# The RNA-binding protein CsrA plays a central role in positively regulating virulence factors in *Erwinia amylovora*

**DOI:** 10.1038/srep37195

**Published:** 2016-11-15

**Authors:** Veronica Ancona, Jae Hoon Lee, Youfu Zhao

**Affiliations:** 1 Department of Crop Sciences, University of Illinois at Urbana-Champaign, Urban 61801, USA

## Abstract

The GacS/GacA two-component system (also called GrrS/GrrA) is a global regulatory system which is highly conserved among gamma-proteobacteria. This system positively regulates non-coding small regulatory RNA *csrB*, which in turn binds to the RNA-binding protein CsrA. However, how GacS/GacA-Csr system regulates virulence traits in *E. amylovora* remains unknown. Results from mutant characterization showed that the *csrB* mutant was hypermotile, produced higher amount of exopolysaccharide amylovoran, and had increased expression of type III secretion (T3SS) genes *in vitro*. In contrast, the *csrA* mutant exhibited complete opposite phenotypes, including non-motile, reduced amylovoran production and expression of T3SS genes. Furthermore, the *csrA* mutant did not induce hypersensitive response on tobacco or cause disease on immature pear fruits, indicating that CsrA is a positive regulator of virulence factors. These findings demonstrated that CsrA plays a critical role in *E. amylovora* virulence and suggested that negative regulation of virulence by GacS/GacA acts through *csrB* sRNA, which binds to CsrA and neutralizes its positive effect on T3SS gene expression, flagellar formation and amylovoran production. Future research will be focused on determining the molecular mechanism underlying the positive regulation of virulence traits by CsrA.

*Erwinia amylovora* is the causal agent of fire blight, a devastating disease of apples and pears, which results in severe economic losses to growers around the world^1^,^2^,^3^. In order to colonize its host and cause disease, *E. amylovora* requires the deployment of effector proteins into the host cells by a type III secretion system (T3SS) and the production of the exopolysaccharide (EPS) amylovoran^4^,^5^,^6^. The T3SS in *E. amylovora* is encoded by the hypersensitive response and pathogenicity (*hrp*) island and is regulated by a HrpL-RpoN sigma factor cascade, which is further activated by the bacterial alarmone (p)ppGpp^7^,^8^,^9^,^10^,^11^,^12^. The T3SS is also controlled by several two-component signal transduction systems (TCST), including GacS/GacA (GrrS/GrrA) and EnvZ/OmpR systems^13^,^14^. On the other hand, amylovoran plays an important role in virulence, biofilm formation, and survival of the bacterium; and its biosynthesis is regulated by the RcsBCD, GrrS/GrrA and EnvZ/OmpR TCST systems^5^,^13^,^14^,^15^.

While widely-distributed among eubacteria, the bacterial CsrA-*csrB*/RsmA-*rsmB* system (for *c*arbon *s*torage *r*egulator/*r*epressor of *s*econdary *m*etabolism, respectively) is a well-characterized and vital small RNA (sRNA)-dependent regulatory system^16^, which regulates a plethora of important phenotypes, including carbon storage, secondary metabolism, motility, biofilm formation, peptide uptake, cyclic di-GMP and (p)ppGpp synthesis, quorum sensing, and expression of virulence genes^17^,^18^,^19^. In many pathogenic bacteria, GacA activates the transcription of one to five small non-coding regulatory RNAs, including *csrB* and *csrC* in *Escherichia coli* and *Salmonella enterica, rsmY* and *rsmZ* in *Pseudomonas aeruginosa, rsmB, rsmY* and *rsmZ* in *Pseudomonas syringae* pv. *tomato* DC3000, and *rsmB* in *Pectobacterium carotovorum* subsp. *carotovorum*^20^,^21^,^22^,^23^,^24^,^25^,^26^. These sRNAs contain many GGA sequences, which are required for sequestering the RNA binding protein CsrA (carbon storage regulator) or its homologs RsmA and RsmE (repressor of secondary metabolites), thus antagonizing its function.

The CsrA protein was first described as a repressor of glycogen metabolism, gluconeogenesis and cell size in *E. coli*^27^. Later studies showed that a *csrA* deletion mutation is not viable in rich medium due to excessive glycogen accumulation^28^. In addition to carbon metabolism, RsmA and RsmE proteins suppress the biocontrol activity of *Pseudomonas protegens* CHA0 by negatively regulating the synthesis of antifungal secondary metabolites^29^. As a major post-transcriptional regulator, CsrA could act both negatively and positively. Transcriptomic, RNA co-purification, and crosslinking and immunoprecipitation (CLIP)-seq data revealed a large posttranscriptional regulon in *Salmonella, E. coli* and *Xanthomonas* spp^30^,^31^,^32^,^33^. Genetic and biochemical studies showed that CsrA-mediated repression primarily affects translation or stability of target mRNAs by blocking ribosome binding through binding to the 5′ untranslated (UTR) regions and recognizing GGA motifs in the apical loops of the RNA secondary structures, one of which overlaps with the Shine-Dalgarno (SD) site^16^,^34^,^35^. Direct binding of CsrA can also stabilize the target mRNAs by masking of RNase E cleavage sites and protecting the transcripts from degradation, or activate translation by enhancing ribosome binding^36^,^37^,^38^. Additionally, CsrA also promotes premature transcription termination by altering Rho-dependent transcript structure^39^.

The Csr/Rsm system has been implicated in regulation of virulence in pathogenic bacteria. In *P. aeruginosa*, an *rsmA* mutation fails to cause actin depolymerization and cytotoxicity in bronchial epithelial cells due to its inability to secrete and translocate T3SS effector proteins^40^. In *Xanthomonas campestris* and *X. oryzae*, RsmA positively regulates exopolysaccharide production and T3SS^41^,^42^. In *X.citri*, RsmA stabilizes mRNA of the master regulator HrpG to activate T3SS^30^. In contrast, in *P. carotovorum*, the *rsmA* mutant was hypervirulent and produced higher levels of cell wall degrading enzymes^40^. In addition, RsmA negatively regulates T3SS in *P. carotovorum* by promoting degradation of the *hrpL* transcript, while *rsmB* is required for the *hrpL* expression^43^. Moreover, *rsmB* enhanced the stability of the *hrpL* transcript in *Dickeya dadantii*, suggesting that RsmA also negatively regulates the *hrpL* gene expression^44^. However, the role of CsrA-*csrB* system in *E. amylovora* has not been elucidated.

The goal of this study was to investigate the role of the CsrA-*csrB* system in *E. amylovora* virulence. Our results provide conclusive evidence that CsrA is a positive regulator of motility, amylovoran production, T3SS and virulence, while *csrB* sRNA, which is under the control of GrrS/GrrA TCST system^13^, negatively regulates these traits.

## Results

### CsrA and *csrB* sRNA from *E. amylovora* are highly conserved

**Figure 1:**
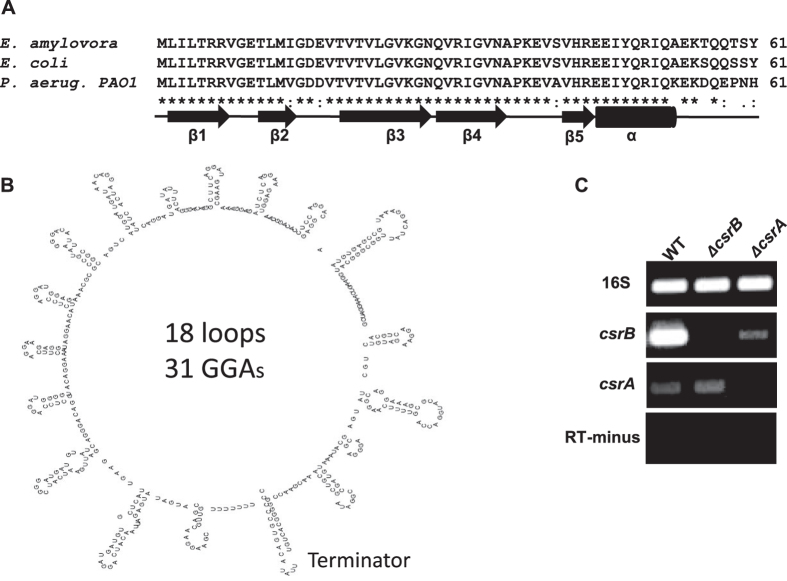
Alignment of deduced amino acids of CsrA and secondary structure of *csrB* from *Erwinia amylovora*. (**A**) Alignment of deduced amino acids of CsrA (accession # CBJ47311 and NP_417176) from *E. amylovora* and *Escherichia coli*; and RsmA (accession #AAG04294) from *Pseudomonas aeruginosa* PAO1, and schematic map of the secondary structure of CsrA based on Schubert *et al*.^56^. Identical residues (*), conserved (:) and semi-conserved (.) substitutions are shown as underneath symbols. (**B**) Predicted secondary structure of *csrB* sRNA using the mfold program; (**C**) Gene expression of *csrB* and *csrA* by semi-quantitative PCR in wild type and the *csrB* and *csrA* mutants in MBMA medium. RT-minus indicates that no mRNA was added.

Analysis of the deduced amino acid sequence of *E. amylovora* CsrA (EAM_ 2637) shows that the 61-amino-acid protein is highly similar to its homologs CsrA from *E. coli* and RsmA from *P. aeruginosa* PAO1, sharing 97 and 85% identity, respectively (Fig. 1A). However, the *csrB* sRNA is not annotated in the *E. amylovora* sequenced genomes. Based on sequences reported in other species^43^,^45^, the *csrB* homolog sequence in *E. amylovora* EA273 genome was located about 200 bases downstream of *EAM_2713* gene and 79 bases upstream of *EAM_2712* gene (Supplementary Fig. S1). The non-coding *csrB* mRNA transcript is 455 nucleotides long and contains 31 GGA motifs, which are essential for CsrA binding^46^,^47^. The RNA folding prediction tool M-Fold predicted that the *E. amylovora csrB* RNA forms 18 loops, which could potentially sequester CsrA (Fig. 1B)^47^. Analysis of the upstream sequence of *csrB* showed that, starting 165 bases from the transcription start site, it contains 18 bp GacA binding site, TGTAAGAGATCGCTT GTA (underlined are conserved), indicating that *csrB* might be regulated by GacA (GrrA)^48^,^49^. An integration host factor (IHF)-binding site (TATCATCTGGTTA) in the upstream region of the *rsmB* sRNA was recently reported in *E. amylovora*^50^, and IHF was required for optimal GacA binding to and transcriptional activation of *csrB* in *E. coli and S. enterica*^51^.

Semi-quantitative PCR was used to determine if CsrA or *csrB* affect each other’s RNA levels (Fig. 1C). As expected, corresponding PCR products were not detected in both mutant strains. The *csrA* RNA levels were not changed in the *csrB* mutant as compared to the wild type (WT) strain. In contrast, the *csrB* RNA levels were decreased about ∼60% in the *csrA* mutant relative to that of the WT strain (Fig. 1C). These results indicate that the *csrB* RNA may not be stable without CsrA protein, suggesting that CsrA is required for the *csrB* RNA accumulation, or that CsrA might indirectly stimulate *csrB* transcription by positively activating GrrA in a feedback regulation^25^,^26^,^52^.

### Growth defect in the *csrA* mutant is independent from glycogen accumulation

During construction of the *E. amylovora csrA* mutant, we observed that the mutant grows very slowly in LB medium. Therefore, we evaluated the growth rate of the *csrA* and *csrB* mutant strains in both LB and MBMA media (Supplementary Fig. S2). The growth rate of the *csrB* mutant strain was similar to that of the WT in LB medium, while growth of the *csrB* mutant was slightly increased in MBMA medium (Supplementary Fig. S2A,B). However, growth of the *csrA* mutant was greatly reduced compared to the WT in LB and MBMA medium, respectively (Supplementary Fig. S2A,B).

Since CsrA suppresses glycogen synthesis in *E. coli*, the excessive accumulation of glycogen impairs growth of a *csrA* deletion mutant and a *glgCAP*/*csrA* double mutation could restore the growth of the *csrA* mutant^28^. Therefore, we examined if a *glgCAP* mutation in *E. amylovora* could also restore the growth of the *csrA* mutant. Growth of the *glgCAP* mutant was similar to the WT in both media, whereas growth of the *csrA*/*glgCAP* double mutant only partially restored the growth of the *csrA* mutant in both media (Supplementary Fig. S2C,D). These results suggest that glycogen accumulation may not be responsible for the slow growth of the *csrA* mutant in *E. amylovora*.

We also determined whether CsrA-*csrB* regulates glycogen accumulation in *E. amylovora*. WT, *glgCAP, csrA*, and *csrB* mutants as well as the *csrB* complementation strain did not accumulate glycogen (Supplementary Fig. S3A)^13^. However, the *csrA* complementation strain showed increased glycogen accumulation, suggesting that in contrary to *E. coli, E. amylovora* CsrA protein positively regulates glycogen synthesis. In addition, these results further confirmed that the growth defect of the *csrA* mutant may not be due to increased glycogen accumulation.

### CsrA and *csrB* sRNA inversely regulate motility

**Figure 2:**
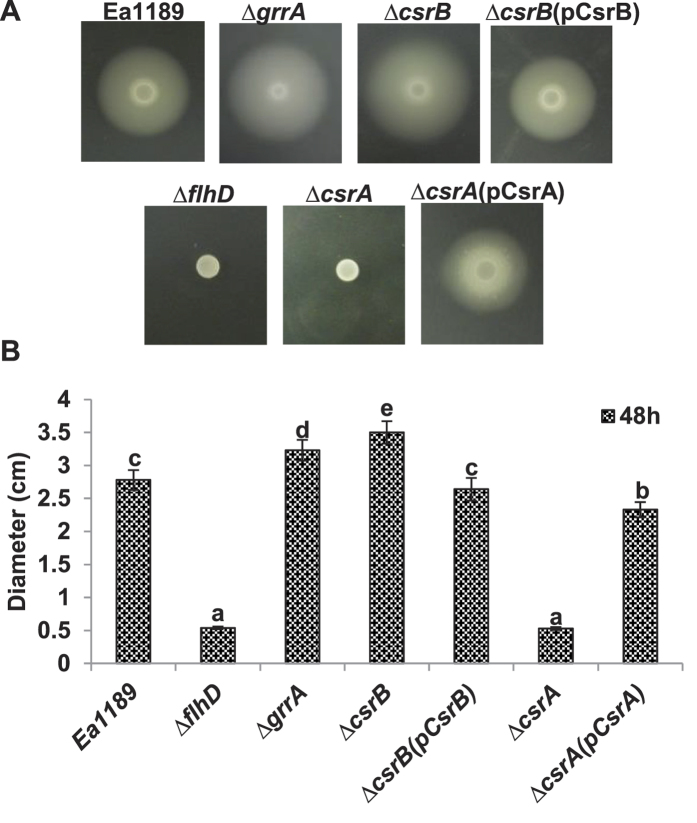
Effect of the *csrA* and *csrB* mutation on motility of *Erwinia amylovora*. (**A**) motility of wild type, the *grrA, flhD, csrA, csrB* mutant and complementation strains on soft tryptone agar plates (3%) at 28 °C. Photographs were taken at 48 h; (**B**) Diameters of the circles were measured 48 h after inoculation.

We have previously reported that mutations on *grrS* /*grrA* gene exhibit increased motility compared to the WT strain^6^,^13^. Measurements of the diameters of the movement circles showed that the *csrB* mutant was hyper-motile similar to that of *grrA* mutant (Fig. 2A,B). In contrast, the *csrA* mutant was non-motile and remained restricted to the plating spot similar to the *flhDC* mutant (Fig. 2A,B). Complementation for the *csrB* and *csrA* mutants restored the motility to the WT levels (Fig. 2A). These results indicated that CsrA and *csrB* sRNA are positive and negative regulators of motility, respectively.

### CsrA and *csrB* sRNA inversely regulate amylovoran production and amylovoran biosynthesis gene expression

**Figure 3:**
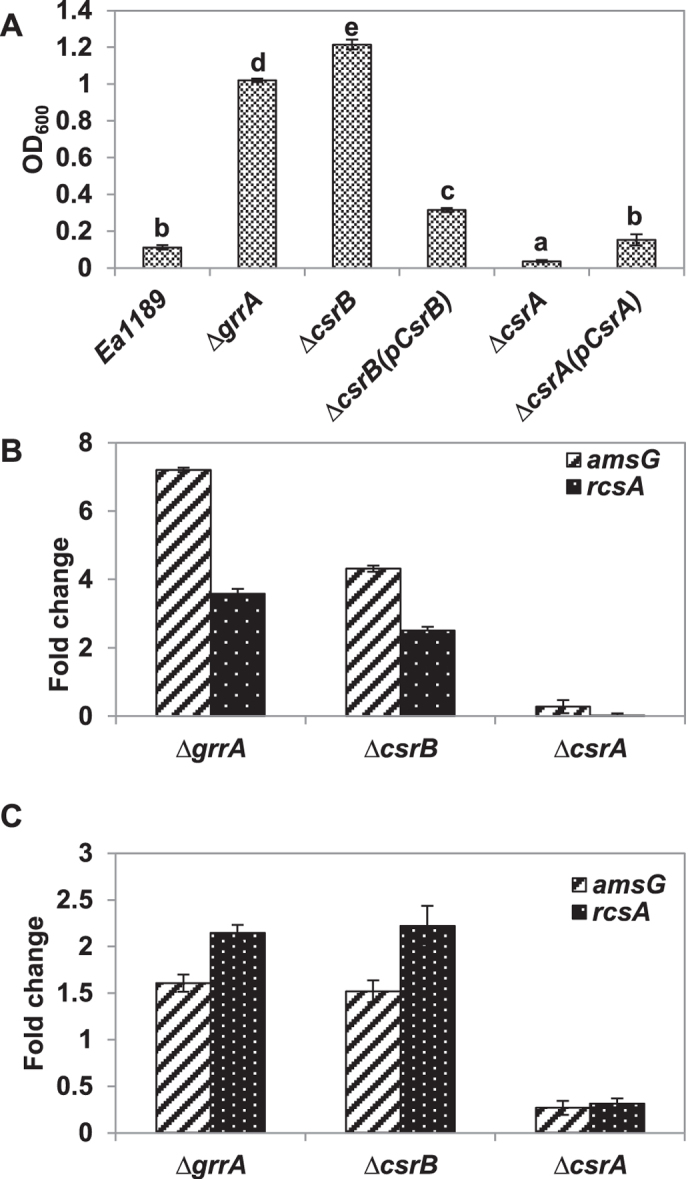
Effect of the *csrA* and *csrB* mutation on amylovoran production and amylovoran biosynthesis and regulatory gene expression. (**A**) Amylovoran production of wild type, the *grrA, csrA, csrB* mutants and complementation strains. Bacterial strains were grown in MBMA media supplemented with 1% sorbitol for 24 h at 28 °C with shaking. Amylovoran concentrations was measured by CPC method and normalized to a cell density of 1. Data points represent the means of three replicates ± standard deviations. OD_600_ = optical density at 600 nm. Each experiment was performed at least two times with similar results. Error bars indicate standard deviations. (**B**) Relative expression of *amsG* and *rcsA* genes in the *grrA, csrB* and *csrA* mutant strains compared to wild type *in vitro* by qRT-PCR. (**C**) Gene expression of *amsG* and *rcsA* genes in the *grrA, csrB* and *csrA* mutant strains as compared to wild type *in vivo*.

We have previously reported that the GrrS/GrrA system negatively regulate amylovoran production *in vitro*^6^,^13^. The amount of amylovoran produced by the *csrB* mutant was ten folds higher than that of the WT after 24 h and similar to that of the *grrA* mutant (Fig. 3A), whereas the *csrA* mutant did not produce any amylovoran (Fig. 3A), which is similar to those of the *rcsB* and *ams* mutants^5^,^6^,^14^. Complementation of the *csrB* and *csrA* mutants partially restored amylovoran biosynthesis, producing approximately 0.31 and 0.15 units of OD_600,_ respectively, as compared to 0.11 units for the WT. Consistent with amylovoran production, relative gene expressions of *amsG* (first gene of the amylovoran biosynthesis operon) and *rcsA* (a rate limit regulatory gene of amylovoran biosynthesis) in the *csrB* and *grrA* mutants were 4–7 and 2.5–3.5 fold higher, respectively, while their expressions were down-regulated 3.5 and more than 10 fold in the *csrA* mutant, respectively, as compared to the WT *in vitro* (Fig. 3B). Similarly, relative expression of *amsG* and *rcsA* genes in the *csrA* mutant was down-regulated 3.7 and 3.1 fold, respectively, as compared to the WT *in vivo* (Fig. 3C). In contrast, *rcsA* and *amsG* gene expression was increased by 2.2 and 1.5 fold *in vivo* in the *csrB* and *grrA* mutants as compared to the WT (Fig. 3C). These results indicated that CsrA is a positive regulator of *rcsA* gene expression and amylovoran production, while *csrB* negatively regulates expression of *rcsA* and amylovoran production.

### Mutation in *csrA*, but not *csrB*, renders *E. amylovora* non-pathogenic and abolishes its ability to elicit HR in tobacco

**Figure 4:**
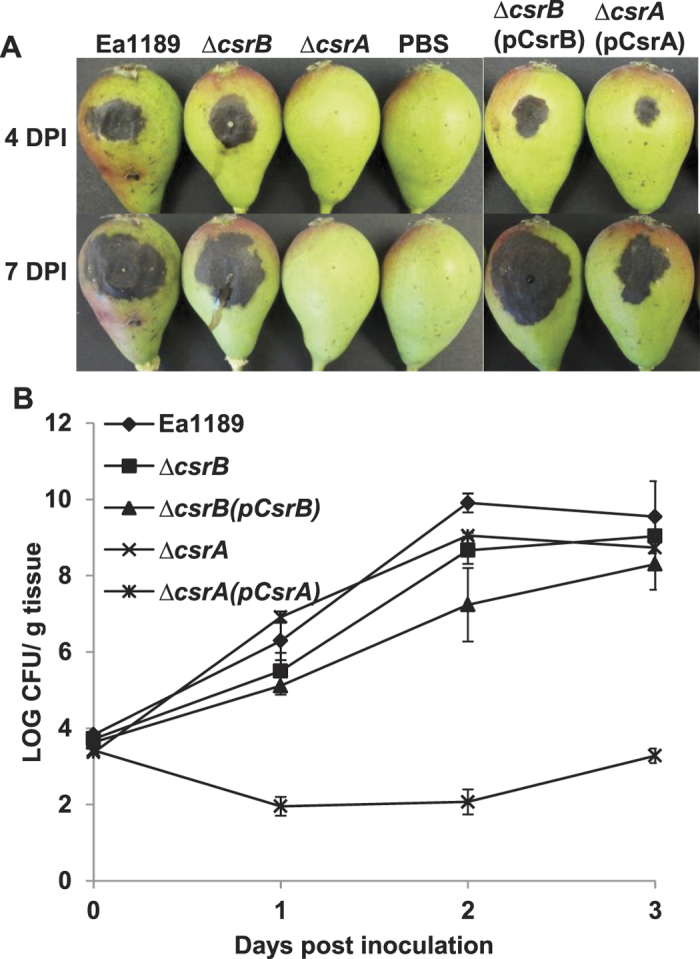
Pathogenicity and growth of *Erwinia amylovora* wild type and mutant strains. (**A,B**) Symptoms caused by wild type, the *csrB* and *csrA* mutants and their complementation strains on immature pear fruits. Immature pears were surface sterilized, pricked with a sterile needle and inoculated with 2 μL of bacterial suspensions. Symptoms were recorded and photos were taken at 4 and 7 days post-inoculation (dpi). (**C**) Growth of *Erwinia amylovora* wild type, mutants and complementation strains. Immature pears were surface sterilized, pricked with a sterile needle and inoculated with 2 μL of bacterial suspensions. Tissue surrounding the inoculation site was excised with a cork borer no.4 and homogenized in 1 mL of 0.5x PBS. Bacterial growth within the pear tissue was monitored after 1, 2 and 3 days post inoculation by dilution plating on LB with appropriate antibiotics.

We then determined the ability of the *csrA* and *csrB* mutants in causing disease on immature pear fruits. For the *csrA* mutant, no symptoms were developed on immature pears, whereas complementation of the *csrA* mutant developed symptoms similar to that of the WT and the *csrB* mutant, but to a lesser degree (Fig. 4A). In addition, the *csrA* mutant was not able to elicit HR in tobacco, whereas WT, the *csrB* and complementation strains did (Supplementary Fig. S3B). These results demonstrated that CsrA, but not *csrB*, is required for causing disease and for eliciting HR in non-host tobacco.

In order to determine whether inability of the *csrA* mutant to cause disease is due to its ability in survival in planta, bacterial growth in pears for the *csrA* and *csrB* mutants were determined (Fig. 4B). At one day post inoculation (DPI), population of the WT, the *csrB* mutant, the *csrB* and *csrA* complementation strains increased from 4 log CFU/g tissue to 6.2, 5.4, 5.1, and 6.9 log CFU/g tissue, respectively. At two and three DPI, the population of these strains reached similar level, between 8–9 log CFU/g tissue. However, population of the *csrA* mutant decreased at one DPI, but gradually increased to 3.2 log CFU/g tissue at three DPI, which was about 6 log difference from that of the WT strain, but similar to the *dspE* and T3SS island deletion mutants^6^,^11^. This result indicated that the *csrA* mutant is able to survive *in planta*, but could not cause disease.

### Expression of T3SS genes is abolished in the *csrA* mutant, but increased in the *csrB* mutant

**Figure 5:**
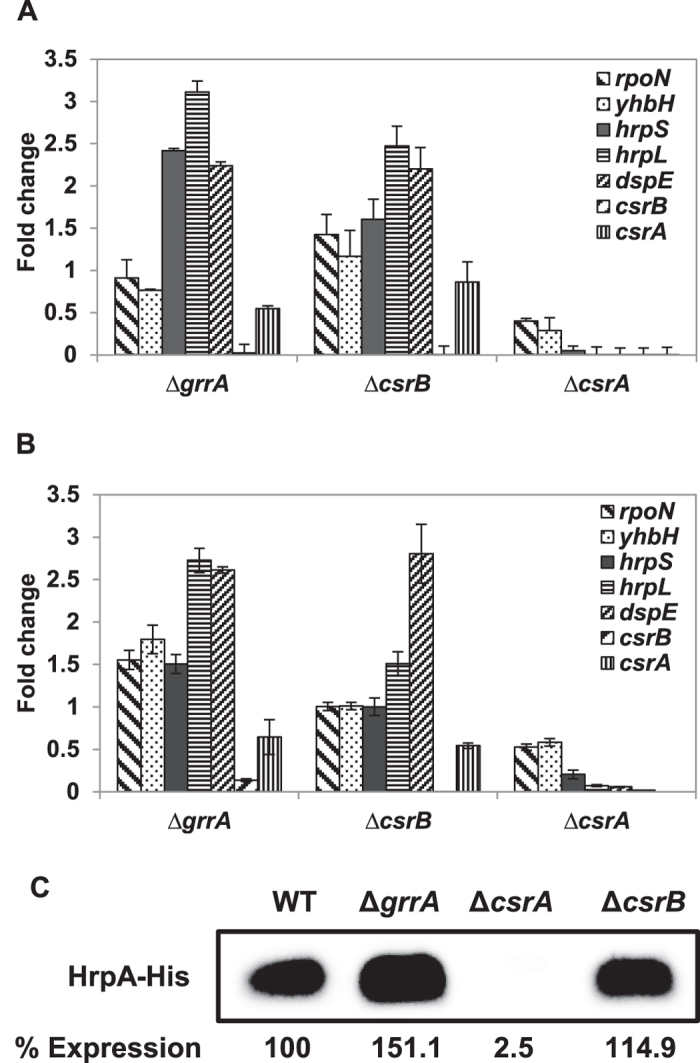
Relative expression of type III secretion genes and accumulation of HrpA protein. Relative expression of T3SS genes in the *grrA,csrB* and *csrA* mutant strains compared to wild type in HMM (**A**) and in pears (**B**). Relative gene expression of selected genes was calculated by the 2^−ΔΔCt^ method, utilizing the 16S rRNA as endogenous control and compared to wild type strain. Fold changes were the result of the mean of three replicates. Each experiment was performed at least two times with similar results. Error bars indicate standard deviations. (**C**) Accumulation of HrpA protein is controlled by CsrA. Abundance of HrpA-His6 protein in WT, the *grrA, csrA*, and *csrB* mutant strains was detected by western blot using anti-histidine protein antibody after grown in *hrp*-inducing minimal medium (HMM) at 18 °C for 6 h. Relative protein abundance was calculated using ImageJ software by utilizing the average pixel value of the signals and utilizing the WT sample as 100%. Cropped blot was displayed and full length blot was presented in Supplementary Fig. S4 as a presentative of results obtained in repeated experiments.

To assess the role of CsrA and *crsB* on the expression of T3SS genes, we compared the relative expression of T3SS regulatory (*rpoN, yhbH, hrpS*, and *hrpL*) and *dspE* effector gene in *grrA, csrB* and *csrA* mutants to those of the WT. Expression of *rpoN* and *yhbH* was not affected in the *csrB* and *grrA* mutants under both *in vitro* and *in vivo* conditions, while expressions of *hrpL* and *dspE* were increased by 2.5–3 and 2.2 folds *in vitro*, and 1.5–2.5 and 2.5–2.8 folds *in vivo*, respectively (Fig. 5A,B). Expression of *hrpS w*as not affected in the *csrB* mutant, but increased about 2.5 fold in the *grrA* mutant (Fig. 5A,B). In the *csrA* mutant, expression of *rpoN* and *yhbH* was down regulated by two folds under both *in vitro* and *in vivo* conditions; whereas expression of *hrpS* was down regulated by five and more than 10 folds under *in vivo* and *in vitro* conditions, respectively. Furthermore, *hrpL* and *dspE* expression was completely abolished in *csrA* mutant under both conditions. These results confirmed that T3SS gene expression is negatively regulated by *csrB* sRNA and positively regulated by CsrA.

Moreover, abundance of HrpA protein in WT and three mutants grown in HMM medium was detected by Western blot (Fig. 5C). Only approximately 2.5% protein signals were detected in the *csrA* mutant, but about 151 and 115% of protein signals were detected in the *grrA* and *csrB* mutants, respectively, as compared to that of the WT strain (Fig. 5C). These results indicate that CsrA is required for T3SS protein accumulation.

We also confirmed the relative expression of the *csrA* and *csrB* genes under the same condition. As expected, *csrA* and *csrB* expression was not detected in the *csrA* or *csrB* mutants, respectively (Fig. 5A,B). Expression of *csrB* was not detected in the *csrA* or *grrA* mutant strains (Fig. 5A,B), confirming the semi-quantitative PCR results that *csrB* is under direct control by GrrA^51^,^53^. In contrast, expression of *csrA* in the *csrB* mutant was not affected under *in vitro* condition, but down-regulated by two folds under *in vivo* condition, whereas expression of *csrA* in the *grrA* mutant was down-regulated by two folds under both conditions (Fig. 5A,B), suggesting CsrA is required for stabilizing *csrB* sRNA and expression of *csrA* is up-regulated in the WT *in vivo*.

## Discussion

The GrrS/GrrA-*csrB*-CsrA system, composed of a two-component system, a non-coding small regulatory RNA *csrB* and a small RNA-binding protein CsrA, is a global dual regulatory system in many pathogenic and saprophytic bacteria^54^,^55^,^56^. Under certain environmental conditions, GrrS/GrrA system specifically activates the expression of *csrB* sRNA, which acts as a “molecular sponge” and binds to CsrA, thus sequestering and antagonizing its function^16^,^47^,^53^,^54^. The CsrA protein, which acts as a translational regulator, binds to target gene mRNA, either altering their translation or stability^17^,^19^. In this study, our results revealed a similar regulatory system exists in *E. amylovora*, and we have provided undeniable evidence that CsrA plays a central role in positively regulating virulence factors, while GrrS/GrrA-*csrB* act as a negative regulators, the latter antagonizing the positive role of CsrA. However, it remains to be determined whether the positive regulatory effect of CsrA on various virulence traits is direct or indirect.

As a global regulator, the GrrS/GrrA-*csrB*-CsrA system conveys pleiotropic effects on multicellular behavior, survival and virulence of various organisms, including glycogen accumulation, secondary metabolism, motility, EPS and enzyme production, T3SS and virulence^17^,^19^,^54^,^57^. As a dual regulator, this system acts as either negative or positive regulator depending on organisms or individual phenotype. However, the global effects of GrrS/GrrA were shown to be mediated by exclusively through its control over transcription of sRNAs in *P. aeruginosa, E. coli* and *S. enterica*^51^,^53^. Optimal GrrA binding to and transcription activation of *csrB* requires IHF in both *E. coli* and *S. enterica*^51^. In *E. amylovora*, mutation of GrrA/GrrS and *csrB* resulted in identical phenotypes, and expression of *csrB* also required IHF^13^,^50^, suggesting that the global effect of GrrS/GrrA might also be exclusively through control over the expression of *csrB* sRNA. Furthermore, the CsrA-*csrB* system in *E. amylovora* works very similarly to those reported in *P. aeruginosa*, where RsmA acts as a central positive regulator and the GrrS/GrrA- *rsmB* acts as a negative regulator^31^; contrary to those reported in other pseudomonads or in closely-related soft rot pathogens such as *Pectobacterium* and *Dickeya*^21^,^44^. Moreover, a *csrA/glgCAP* double mutant could not restore the slow growth of the *csrA* mutant in *E. amylovora* as it does in *E. coli*^28^, which may be due to that in *E. amylovora*, glycogen accumulation appears to be mainly regulated by another global regulator, the EnvZ/OmpR system^13^.

CsrA-mediated negative posttranscriptional regulation normally involves CsrA protein binding to the 5’ UTR or initially translated region of target mRNAs, which contains multiple CsrA-binding sites (GGA) and one overlapping with SD sequence^47^. Bound CsrA thus represses translation by competing with ribosome binding, leading to destabilization of the target mRNAs^17^,^46^. CsrA also can activate translation or stabilize target mRNAs by enhancing ribosome binding or by protecting the transcripts from RNase E-dependent degradation^36^,^37^. In *E. amylovora*, CsrA positively regulates motility as reported in *E. coli* and *P. aeruginosa*; and in contrast to those in *Pectobacterium wasabiae* and *P. carotovorum*^38^,^58^,^59^. It has been reported that CsrA in *E. coli* binds to two sites of the *flhDC* leader sequence and stabilizes the *flhDC* transcript by masking of the RNase E cleavage sites and protecting the transcript from degradation^37^. It is most likely that *E. amylovora* CsrA utilizes a similar mechanism to stabilize and protect the *flhDC* transcripts, which needs to be further proven.

Besides motility, CsrA also positively regulates both amylovoran production and T3SS in *E. amylovora*. The key question remaining is the underlying molecular mechanism of how CsrA positively regulates these virulence traits in *E. amylovora*. In *E. amylovora*, amylovoran biosynthesis is positively regulated by the RcsBCD phosphorelay system and RcsA, the rate limiting factor^5^,^60^; whereas GrrS/GrrA, EnvZ/OmpR, Lon protease, global regulator H-NS, and orphan protein AmyR (YbjN) are all known negative regulators of amylovoran production^5^,^13^,^14^,^60^,^61^,^62^,^63^. H-NS binds to the promoter of *rcsA* and suppresses its expression; whereas the RcsA protein is subject to Lon-dependent degradation. Additionally, AmyR/YjbN was characterized as a novel negative regulator of EPS production in both *E. coli* and *E. amylovora* and may act as a protein stabilizer^62^,^63^. Recent studies have shown that both *lon* and *ybjN* mRNA could be co-purified with CsrA protein in *E. coli*^32^, but CsrA only bound to the coding sequence of *lon* mRNA^33^. In this study, expression of *rcsA* gene was almost abolished in the *csrA* mutant. Therefore, further studies are needed to determine the molecular targets of CsrA in regulating amylovoran production.

In *E. amylovora*, transcription of the T3SS genes is positively activated by (p)ppGpp-mediated alternative sigma factor cascade, including the master regulator HrpL, alternative sigma factor RpoN, enhancer binding protein (EBP) HrpS, ribosome-binding protein YhbH, IHF, transcriptional factor DksA and (p)ppGpp biosynthesis proteins RelA and SpoT^7^,^8^,^10^,^64^; whereas GrrS/GrrA and EnvZ/OmpR systems negatively regulates T3SS gene expression^13^. In *P. carotovorum*, expression of *hrpL* is positively regulated by GacA/GacS-*rsmB* system, while RsmA negatively controls its expression^43^. Positive regulation of T3SS by RsmA is also reported in *P. aeruginosa, X. campestris* and *X. citri*^30^,^31^,^40^,^41^. It has been reported that RsmA activates T3SS by stabilizing the 5′ UTR of HrpG mRNA in *Xanthomonas*, suggesting that RsmA may regulate T3SS gene expression through HrpG, the master regulator of T3SS^30^. In addition, (p)ppGpp-mediated stringent response and CsrA regulons shared extensive overlap, suggesting that regulatory interaction exists between CsrA and (p)ppGpp-mediated stringent response regulatory system^17^,^32^. Therefore, CsrA -dependent control of T3SS gene expression via major transcription regulatory factors might be a conserved feature among pathogenic bacteria^19^. In this study, our results showed that CsrA is required for expression of T3SS genes in *E. amylovora*, including *rpoN, yhbH, hrpS* and *hrpL* regulatory genes, suggesting that these major regulatory genes might be potential targets of CsrA in *E. amylovora*. Additionally, a *lon* mutation in *P. syringae* increases the stability and accumulation of HrpR, another EBP similar to HrpS^65^,^66^. Therefore, future studies are needed to uncover the molecular mechanisms as how CsrA positively regulates T3SS in *E. amylovora*.

**Figure 6:**
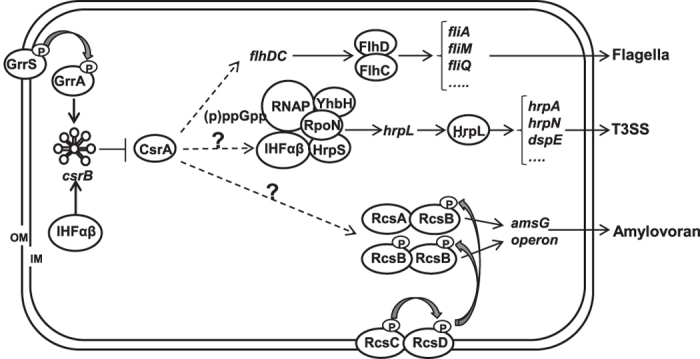
A working model illustrated the role of the RNA-binding protein CsrA in *Erwinia amylovora*. This model is based on findings obtained in this study as well as those reported in previously studies^7^,^8^,^9^,^10^,^13^,^14^,^50^,^60^. FlhDC: master regulator of flagellar formation; HrpL: an ECF sigma factor and master regulator of T3SS; HrpS: a σ^54^-dependent enhancer binding protein; Ihfα/β: integration host factor αβ; RpoN: a σ^54^ alternative sigma factor; YhbH: σ^54^ modulation protein (ribosome-associated protein); RNAP: RNA polymerase. (p)ppGpp: guanosine tetraphosphateand guanosine pentaphosphate; GrrS/GrrA; RcsABCD: two-component regulatory systems; *csrB*: small non-coding regulatory RNA; CsrA: RNA-binding protein; OM, outer membrane; IM, inner membrane; P, phosphorylation. Symbols: ↓, positive effect; ⊥, negative effect; dash line with/without ?: unknown mechanism.

Based on our results and previous reported data^7^,^8^,^9^,^10^,^13^,^14^,^50^,^60^, we proposed the following working model as how the GrrS/GrrA-*csrB*-CsrA system might regulate virulence traits in *E. amylovora* (Fig. 6). In *E. amylovora*, the T3SS is activated by (p)ppGpp-mediated RpoN-HrpL sigma factor cascade; whereas amylovoran biosynthesis is positively regulated by the RcsABCD TCST system. In addition, the global GrrA/GrrS TCST along with integration host factor (IHF) specifically regulates the expression of *csrB* sRNA, which acts as a “sponge” to antagonize the effect of CsrA^13^,^25^,^49^,^50^,^51^,^53^. As discussed above, CsrA is a positive regulator of motility in *E. amylovora* possibly by stabilizing and protecting *flhDC* transcript from RNase E degradation as previously reported^37^. However, the targets of CsrA in positively regulating T3SS and amylovoran biosynthesis remain unknown. Currently, we are focusing on uncovering the underlying molecular mechanism of CsrA as a central positive regulator of virulence factors in *E. amylovora*.

## Methods

### Bacterial strains and growth conditions

Bacterial strains and plasmids used in this study are listed in Table 1. LB broth was used for routine growth of *E. amylovora* and *E. coli* strains. For amylovoran production assays, bacteria were grown in MBMA medium (3 g KH_2_PO_4_, 7 g K_2_HPO_4_, 1g [NH_4_]_2_SO_4_, 2 mL glycerol, 0.5 g citric acid, 0.03 g MgSO_4_) supplemented with 1% sorbitol^5^. A *hrp*-inducing minimum medium (HMM) (1g [NH_4_]_2_SO_4_, 0.246 g MgCl_2_•6H_2_O, 0.1 g NaCl. 8.708 g K_2_HPO_4_, 6.804 g KH_2_PO_4_) with 10 mM galactose as carbon source was used for inducing T3SS gene expression^8^,^67^. When required, antibiotics were used at the following concentrations: 50 μg mL^−1^ Kanamycin, 100 μg mL^−1^ Ampicillin and 10 μg mL^−1^ chloramphenicol. Primers used for mutant construction, mutant confirmation, RT-qPCR and cloning in this study are listed in Table S1.

### Generation of single and double mutants by λ–Red recombinase cloning

*E. amylovora* mutant strains were generated using λ phage recombinase method as describe previously^6^,^68^. Briefly, overnight cultures of *E. amylovora* strains harboring pKD46 were inoculated in LB broth containing 0.1% arabinose and grown to exponential phase (OD_600_ = 0.8). Cells were harvested, made electrocompetent and stored at −80 °C. These cells were electroporated with recombination fragments of *cat* or *kan* genes with their own promoter. *Cat* and *kan* fragments were obtained by PCR amplification from pKD32 or pKD13 plasmids, respectively, flanked by a 50-nucleotide homology from the target genes. To confirm *csrA, csrB*, and *glgCAP* mutations, PCR amplifications from internal *cat* or *kan* primers to the external region of the target genes were performed. The coding region of the *csrA, csrB*, and *glgCAP* genes was absent from the corresponding mutant strains, except for the first and last 50 nucleotides.

### Construction of the *csrA* and *csrB* complementation plasmids

For complementation of the mutant strains, the genomic region containing the promoter and gene sequence of *csrA* and *csrB* were PCR amplified, gel purified and cloned into pGEM-T-easy vector according to manufacturer’s instructions (Promega, WI). Plasmid DNA purification, PCR amplification of genes, isolation of fragments from agarose gels were performed using standard molecular procedures^69^. Plasmid verification was performed by sequencing at the UIUC Core Sequencing Facility. Final plasmids were designated as pCsrA and pCsrB and transformed by electroporation into corresponding mutant strains.

### Motility assay

Bacterial strains were grown overnight in LB with appropriate antibiotics, harvested by centrifugation and washed three times with PBS. Bacterial suspensions were adjusted to an OD_600_ equal to 1.0, and 5 μL drops were placed onto the center of agar plates (10 g tryptone, 5 g NaCl, 3 g agar per liter) as previously described^14^. Plates were incubated at 28 °C and movement diameters were measured after 48 h post inoculation. The experiments were performed in triplicate and repeated at least three times.

### CPC assay to determine amylovoran concentration

Amylovoran concentration of the WT, mutant and complementation strains was quantitatively determined by the cetylpyrimidinium chloride (CPC) method as previously described^70^. Briefly, overnight cultures of bacterial strains were harvested by centrifugation and washed three times with PBS. Five mL MBMA medium supplemented with 1% sorbitol were inoculated to a final OD_600_ of 0.2 and incubated at 28 °C with shaking. After 24 h, 1 mL of each culture was centrifuged at 7,000 rpm for 10 min and 50 μL of CPC at 50 mg mL^−1^ was added to the supernatant. After 10 min of incubation, the turbidity of the suspension and cell density was determined by measuring OD_600_. Amylovoran was determined by normalizing the OD_600_ of the suspension to a cell density of 1.0. Each experiment was performed in triplicate and repeated at least three times.

### RNA isolation

Bacterial strains grown overnight in LB media with appropriate antibiotics were harvested by centrifugation and washed three times with 0.5X PBS before inoculating 5 mL of MBMA medium supplemented with 1% sorbitol. For *hrp*-inducing conditions, bacterial cells were washed three times with HMM before inoculated 5 mL of medium to a final OD_600_ = 0.2. After 18 h incubation for MBMA at 28 °C or 6 h incubation for HMM at 18 °C, 2 mL of RNA protect reagent (Qiagen) was added to 1 mL of bacterial cell culture mixed by vortex and incubated at room temperature for 5 min. Cells were harvested by centrifugation and RNA was extracted using RNeasy^®^ mini kit (Qiagen) according to the manufacturer’s instructions. DNase I treatment was performed in column before elution and RNA was quantified using Nano-drop ND100 spectrophotometer. For *in vivo* conditions, overnight cultures of bacterial strains were harvested by centrifugation, washed three times and suspended in PBS. Immature pear fruits were cut in half and inoculated with bacterial suspensions. After 6 h incubation at 28 °C in a moist chamber, bacterial cells were collected by washing pear surfaces with RNA protect reagent (Qiagen) 2:1 with water and total RNA was extracted as described above.

### Reverse transcription quantitative real-time PCR (qRT-PCR)

One microgram of total RNA was reverse transcribed using Superscript III reverse transcriptase (Invitrogen, Carlsbad, CA) following the manufacturer’s instructions. One μL of cDNA was used as template for qPCR performed using the ABI 7300 System (Applied Biosystems, Foster City, CA). Power SYBR^®^ Green PCR master mix (Applied Biosystems) was used to detect gene expression of selected genes with primers designed using Primer3 software. qPCR amplifications were carried out at 50 °C for 2 min, 95 °C for 10 min, followed by 40 cycles of 95 °C for 15 s and 60 °C for 1 min. Dissociation curve analysis was performed after the program was completed to confirm amplification specificity. Three technical replicates were performed for each biological sample. Relative gene expression levels were calculated with the 2^−∆∆Ct^ method using the 16s rRNA (*rrsA*) as endogenous control and wild type as reference value.

### Semi-quantitative RT-PCR

Total RNA from MBMA medium was extracted and reverse transcribed as described above. PCR reactions were performed on 10 ng of cDNA using *csrA*-rt and *csrB*-rt and 16S-rt primers listed in Table S1. RNA samples were used as template for RT-minus controls. PCR amplification was carried out at 94 °C for 2 min, followed 20 cycles of 94 °C for 30 s, 60 °C for 30 s and 72 °C for 30 s. PCR products were separated on an ethidium bromide pre-stained 1% agarose gel, and visualized in a Molecular Imager^®^ Gel Doc™ XR System (Bio-Rad).

### Virulence assays on immature pear fruits

Virulence assays were performed as described previously^11^,^12^. Briefly, overnight cultures of *E. amylovora* WT, mutants and complementation strains were harvested by centrifugation and suspended in 0.5x PBS. Immature pear fruits (*Pyrus communis* L. cv. ‘Bartlett’) were surface sterilized with 10% bleach, pricked with a sterile needle and inoculated with 2 μL of 100 X dilution of bacterial suspensions at OD_600_ = 0.1. Inoculated pears were incubated at 28 °C in a humidity chamber and disease symptoms were recorded after 4 and 7 days post inoculation. The experiment was performed in triplicate at least three times. For bacterial population studies, pears were inoculated as described above and the tissue surrounding the inoculation site was excised with a cork borer no.4 and homogenized in 1 mL of 0.5x PBS. Bacterial growth within the pear tissue was monitored after 1, 2 and 3 days post inoculation by dilution plating on LB with appropriate antibiotics^11^,^12^. For each time point and strain tested, fruits were assayed in triplicate. The experiment was performed three times.

### Western blot

*E. amylovora* cells grown in HMM at 18 °C for 6 h were harvested, and equal amount of cell lysates was separated by sodium dodecyl sulfate polyacrylamide gels^8^. Proteins were transferred to polyvinylidene fluoride membrane (Millipore) and blocked with 5% milk in phosphate-buffered saline (PBS). To detect HrpA-6His, membranes were probed with rabbit anti-His antibodies (GeneScript, Piscataway, NJ) that were diluted to 1.0 μg/ml with PBS containing 0.1% Tween 20 (PBST). Immunoblots were then developed with horseradish peroxidase-linked anti-rabbit IgG antibodies (Amersham Biosciences) diluted 1: 10,000 in PBST, followed by enhanced chemiluminescence reagents (Pierce). Images of the resulting blots were acquired using ImageQuant LAS 4010 CCD camera (GE Healthcare). The experiment was performed at least three times.

### Statistical analysis

One-way ANOVA and Student-Newmans-Kleus test (p = 0.05) was used to analyze the data. For WT, mutants and complementation strains, changes marked with the same letter did not differ significantly (P < 0.05).

## Additional Information

**How to cite this article**: Ancona, V. *et al*. The RNA-binding protein CsrA plays a central role in positively regulating virulence factors in *Erwinia amylovora. Sci. Rep.* **6**, 37195; doi: 10.1038/srep37195 (2016).

**Publisher’s note:** Springer Nature remains neutral with regard to jurisdictional claims in published maps and institutional affiliations.

## Supplementary Material

Supplementary Information

## Figures and Tables

**Table 1:** Bacterial strains and plasmids used in this study.

Strains or plasmids	Description	Reference or source
*E. amylovora*		
Ea1189	Wild type, isolated from apple	71
Z2946∆*flhD*	*flhD*::Km; Km^R^-deletion mutant of *flhD* of Ea1189, Km^R^	14
∆*csrA*	*csrA*::Cm; Cm^R^-deletion mutant of *csrA* of Ea1189, Cm^R^	This study
∆*csrB*	*csrB*::Cm; Cm^R^-deletion mutant of *csrB* of Ea1189, Cm^R^	13
∆*glgCAP*	Cm^R^-deletion mutant of *glgCAP* operon (5.2 kb) of Ea1189, Cm^R^	This study
∆*csrA/glgCAP*	*csrA*::Cm, *glgCAP*::Km; Km^R^-deletion mutant of *glgCAP* into ∆*csrA*	This study
Z2198∆*grrA*	*grrA*::Km; Km^R^-deletion mutant of *grrA* of Ea1189, Km^R^	14
DH10B *E. coli strain*	F– *mcr*A Δ(*mrr*-*hsd*RMS-*mcr*BC) Φ80*lac*ZΔM15 Δ*lac*X74 *rec*A1 *end*A1 *ara*D139 Δ(*ara leu*) 7697 *gal*U *gal*K *rps*L *nup*G λ	Invitrogen, CA
Plasmids		
pKD46	Ap^R^, P_BAD_ *gam bet exo* pSC101 oriTS	68
pKD32	Cm^R^, FRT *cat* FRT tL3 oriR6Kγ *bla rgnB*	68
pkD13	Km^R^, FRT *kan* FRT tL3 oriR6Kγ *bla rgnB*	68
pHrpA-His6	803-bp DNA fragment containing promoter sequence of the *hrpA* gene and c-terminal His tag in pWSK29	8
pGem^®^T-easy	Ap^R^, PCR cloning vector	Promega WI
pCsrA	A 824 bp fragment containing *csrA* gene in pGemT-easy vector	This study
pCsrB	A 1.3-kb fragment containing *csrB* in pGemT-easy vector	This study
