# Supplementary material for: The RNA-binding protein CsrA plays a central role in positively regulating virulence factors in Erwinia amylovora
Source: Sci Rep. 2016 Nov 15;6:37195. doi: 10.1038/srep37195 (PMC5109040; doi:10.1038/srep37195)
Supplement: Supplementary Information [file srep37195-s1.pdf]

The RNA-binding protein CsrA plays a central role in positively regulating virulence factors in *Erwinia amylovora*

Veronica Ancona[1, $], Jae Hoon Lee[1], and Youfu Zhao[1, *]

Supplementary Figure S1. Sequence of *csrB* sRNA.

Supplementary Figure S2. Growth of the *csrA, csrB, glgCAP* and *csrA/glgCAP* double mutants of *Erwinia amylovora*. Growth of wild type, mutants and complementation strains in LB (A and C) and MBMA (B and D) medium measured by spectrometry at 600 nm. The experiments were repeated three times with three replicates.

Supplementary Figure S3. A) Effect of the *csrA* and *csrB* mutations on glycogen accumulation. Overnight cultures of the WT, mutants and complementation strains were centrifuged, suspended in 0.5 X PBS and spotted onto Kornberg medium (1.1% K_2HPO_4, 0.85% KH_2PO_4, 0.6% yeast extract, 1% glucose) agar plates. After 24 h incubation at 28°C, the plates were exposed to iodine vapor for 10 min to assess glycogen accumulation[1,2]. The experiments were repeated at least three times. B) **Hypersensitive response (HR) assay on tobacco leaves**. Photos were taken at 24h post inoculation. Overnight cultures of *E. amylovora* WT, mutants and complementation strains were harvested by centrifugation and suspended in 0.5x PBS to OD_{600} of 0.1. Bacterial suspensions were infiltrated into tobacco leaves (*Nicotiana tabaccum*) by needle-less syringe. HR symptoms were recorded after 24h post infiltration. HR experiments were repeated three times.

Reference:

1. Ancona, V., Li, W.& Zhao, Y. F. Alternative sigma factor RpoN and its modulator protein YhbH are indispensable for Erwinia amylovora virulence. Mol. Plant Pathol. 15,58-66 (2014).
2. Romeo, T., Kumar, A. & Preiss, J. Analysis of the *Escherichia coli* glycogen gene cluster suggests that catabolic enzymes are encoded among the biosynthetic genes. *Gene* **70**, 363–376 (1988).

Supplementary Figure S4. Full-length Western blot for HrpA-His was presented. See Figure 5 for details.

Fig. S1. *csrB* sRNA coding sequence (position 2955579 to 2956033)

```
Genome accession #FN666575 for E. amylovora strain ATCC49946

GTTGCGAAGGAACAGCATGATATGAGATTAACATCAGGATGATGTGCTCATTGAAGGATTGAAT
CATCGGGATGATGTATCAGGGACAGGCTCCAGGATGGGGTACAGGAAACTTCAGGAAGAGGTCA
GGAACATCTCCAGGATGGAGAAACGCGCCGTAAGGGACATTCGGCAGTCATGGATGATAACAGG
ATCACTATCAGGATGATATTTCAGGGACAGGCTTCAGGATGAAGCAAAGGAGATCTCAGGAAGA
GGTAATGGACACCTCCAGGACGGAGAATGTGAGCCGTAAAGGACTATTGGCGGGCATGGAAGTC
AAAGGATCGACGTCAGGAAGATGTCTGCTCAGGAAAGCGCATGGACCAGTTTTCAAGGATGAGC
AGGGAGCATAAATGTAGCCGGATAGCTGCAAACGAACCGGGGGCACTGTTTATACAGTGCCCCC
TTTTTTT
```

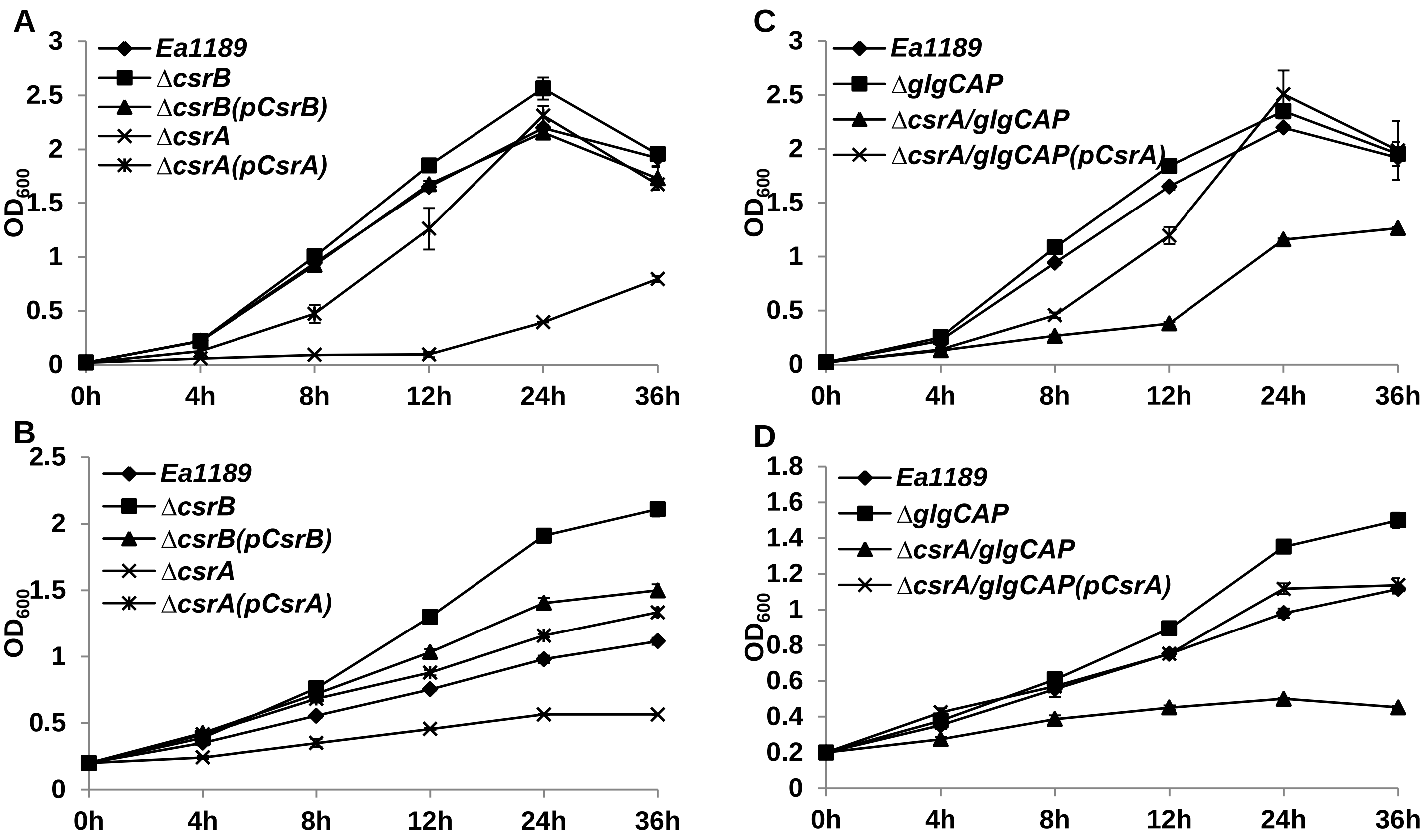

Ancona et al. Fig. S2

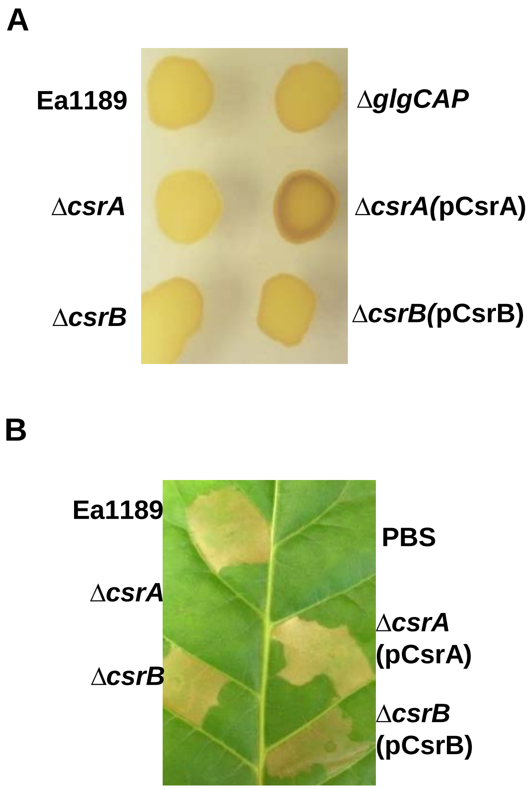

Ancona et al. Fig. S3

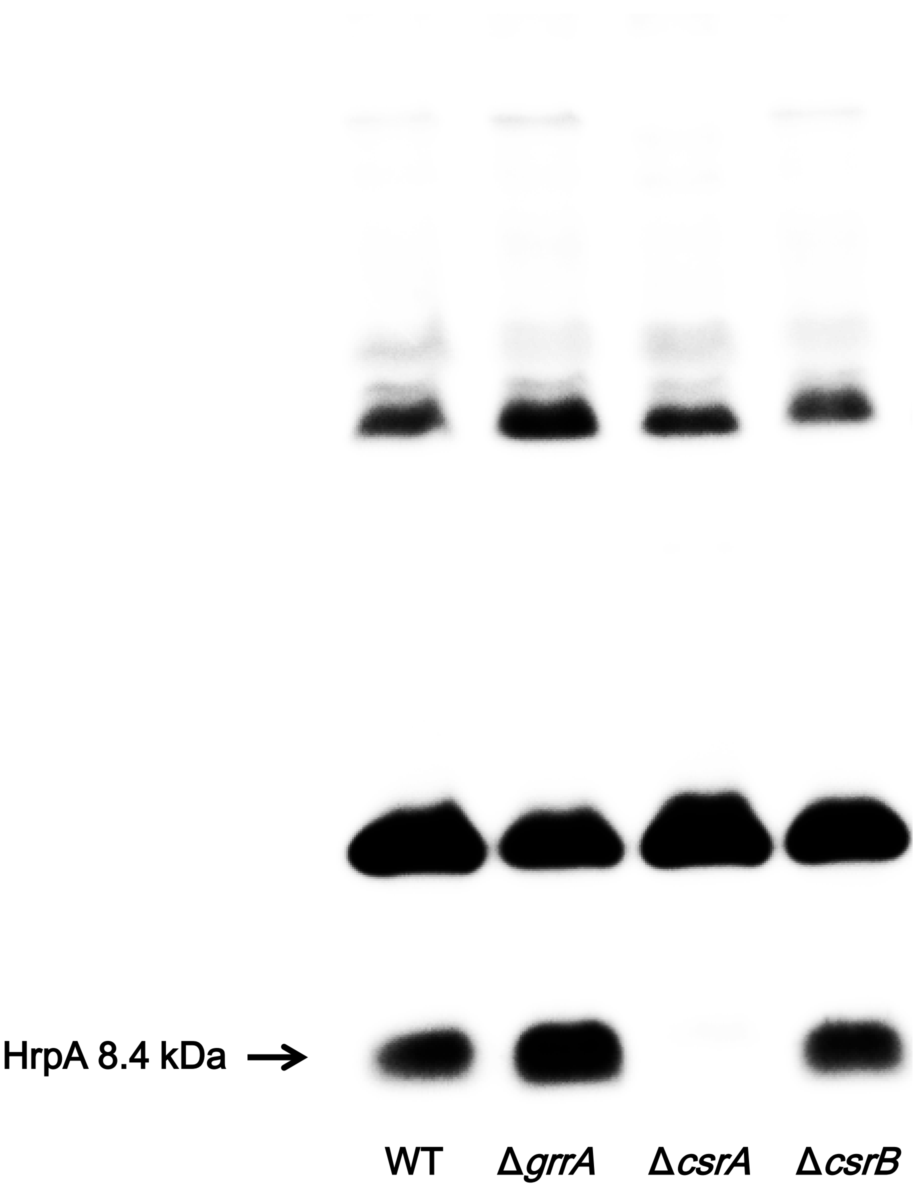

Ancona et al. Fig. S4

Supplementary Table S1. List of primers used in this study

Primers	Sequence (5'-3')	Source or reference
Mutagenesis		
csrA-F	ATGCTTATTCTAACTCGTCGAGTTGGTGAAACCCTCATG ATCGGTGATGACGATTGTGTAGGCTGGAGCT	This study
csrA-R	TTAGTAACTCGTTTGCTGCGTCTTTTCGGCCTGTATACGC TGATAGATCTATTCCGGGGATCCGTCGACC	This study
csrB-F	GTTGCGAAGGAACAGCATGATATGAGATTAACATCAGG ATGATGTGCTCAGCGATTGTGTAGGCTGGAGCT	This study
csrB-R	AAAAAAAGGGGGCACTGTATAAACAGTGCCCCCGGTTC GTTTGCAGCTATATTCCGGGGATCCGTCGACC	This study
glgCAP-F	ATGGTGAAATTAGAGAAGAACGACCCTGTGATGCTGGC AAGGCAATTACCCGATTGTGTAGGCTGGAGCT	This study
glgCAP-R	CTATAACTGAACCGGCTCAATACCCCAAATTTCGTCGGC ATATTCTTTGAATTCCGGGGATCCGTCGACC	This study
csrA-c1	GGTACGGATGCAATGGCTTT	This study
csrA-c2	TGCCTAAACCAGCTTAATGGA	This study
csrB-c1	GCCTGCCCTGTACGAGAT	This study
csrB-c2	GGCCTGTTTGAGTAACGAT	This study
glgCAP-c1	ACGTGTGCGATCTGGTGTTA	This study
glgCAP-c2	TCCTGGATTCATAGCCAGAC	This study
Cm1	TTATACGCAAGGCGACAAGG	14
Cm2	GATCTTCCGTCACAGGTAGG	14
Cloning		
csrA-coF	GTACGGATGCAATGGCTTT	This study
csrA-coR	TGCCTAAACCAGCTTAATGGA	This study
csrB-coF	CGCGTTCTATACTTCTCAGTTCG	This study
csrB-coR	CTCCAGCGCGACTTCATAAT	This study
Real time PCR		
csrA-rt1	TCATGATCGGTGATGAGGTG	This study
csrA-rt2	ACTCGTTTGCTGCGTCTTTT	This study
csrB-rt1	CCTGACGTCGATCCTTTGAC	This study
csrB-rt2	GTAAGGGACATTCGGCAGTC	This study
rcsA-rt1	TTAAACCTGTCTGTGCGTCA	67
rcsA-rt2	AGAAACCGTTTTGGCTTTGA	67
amsG-rt1	CAAAGAGGTGCTGGAAGAGG	67
amsG-rt2	GTTCCATAGTTGCGGCAGTT	67
rpoN-rt1	AAGCGGTACTGAAACGGGTA	7
rpoN-rt2	GCATCAGACTGCGAAAATCA	7
yhbH-rt1	GCGCGAGTTTGTTACCACTA	7
yhbH-rt2	ATCGCCGCGTACATATCTTT	7
hrpS-rt1	AACAATGGCGTTTGCGTTGC	7
hrpS-rt2	AATGCTACGCGTGCTGGAAA	7
hrpL-rt1	TTAAGGCAATGCCAAACACC	7
hrpL-rt2	GACGCGTGCATCATTTTATT	7
dspE-rt1	TCCAGCGAGGGCATAATACT	67
dspE-rt2	ACAACCGTACCCTGCAAAAC	67
16S-rt1	TGTAGCGGTGAAATGCGTAG	67
16S-rt2	CCTCCAAGTCGACATCGTTT	67
